# Shock-Induced Degradation of Guanosine and Uridine Promoted by Nickel and Carbonate: Potential Applications

**DOI:** 10.3390/molecules28248006

**Published:** 2023-12-08

**Authors:** Gustavo P. Maia, José Armando Luísa da Silva, Vânia André, Adelino M. Galvão

**Affiliations:** Centro de Química Estrutural, Institute of Molecular Sciences, Departamento de Engenharia Química, Instituto Superior Técnico, Universidade de Lisboa, Av. Rovisco Pais, 1, 1049-001 Lisboa, Portugal; gustavo.pinho.maia@tecnico.ulisboa.pt (G.P.M.); vaniaandre@tecnico.ulisboa.pt (V.A.)

**Keywords:** mechanochemistry, DFT, degradation reactions, meteorites, asteroids

## Abstract

Experimental studies of the degradation of two ribonucleosides (guanosine and uridine) were carried out by making use of mechanochemistry. Mechanochemical experiments reveal the decomposition of guanosine and uridine, promoted by nickel(II) and carbonate ions, into guanine and uracil, respectively. These nucleobases were identified by HPLC and ^1^H NMR spectroscopy (this applied only to uracil). Additionally, density-functional theory (DFT) methodologies were used to probe the energetic viability of several degradation pathways, including in the presence of the abovementioned ions. Three mechanisms were analysed via ribose ring-opening: dry, single-molecule water-assisted, and metal-assisted, wherein the last two mechanisms confirmed the mechanochemical degradation of both ribonucleosides into respective nucleobase moieties. These results can contribute to an astrobiological interpretation of the extraterrestrial sample’s contents.

## 1. Introduction

Mechanochemistry makes use of the absorption of mechanical energy (by molecules, ions, etc.) to promote chemical reactions [1]. Depending on the type of ball milling (mixer, planetary, or rolling mill), several sources of mechanical energy (impact, tension, and friction) can take place [2]. Shear force and non-hydrostatic compression forces are two major examples present in mechanochemical experiments [3]. Despite its applicability in a variety of fields, as in green synthetic methods [2], mechanochemistry could also be applied to the degradation of organic molecules [2,4,5]. Polymer degradation and stability studies of active pharmaceutical compounds are two major fields where mechanochemically induced degradation can be applied [4,6,7]. Many compounds contain a mechanophore, which is a portion of a molecule that follows a known mechanical perturbation [8], which could help in the elucidation of degradation mechanisms, for example, for biomolecules. Homolytic, heterolytic, and coordinated bond cleavage are three types of known chemical degradation patterns present in mechanophores [8]. However, until now, it has not been proven that covalent bond-based mechanophores are present in biological systems as opposed to non-covalent ones [9].

In modern biology, ribonucleosides are an important portion of the building blocks of ribonucleic acid (RNA). Their stability is directly related (although not exclusively) to the *N*-glycosidic bond and differs from canonical purine (guanosine and adenosine) and pyrimidine (cytidine and uridine) ribonucleosides. For instance, ribonucleoside thermal stability is proportional to *N*-glycosidic bond order, wherein guanosine and cytidine are less stable than adenosine and uridine [10,11]. Ribonucleoside photostability is also related to the *N*-glycosidic bond, and despite their photoresistance under energetic beams, guanosine and adenosine appear to endure more compared with cytidine and uridine [12]. Ribonucleosides can also undergo acid-/base-catalysed *N*-glycosidic hydrolysis; in this case, canonical pyrimidine ribonucleosides are more stable than purine moieties [13]. For the majority of the ribonucleoside degradation pathways abovementioned, the major degradation product is the corresponding purine/pyrimidine nucleobase. This implies that the main degradation mechanism consists of ribose decomposition. In fact, some inorganic ions could also promote the degradation of ribose as carbonate, while other ions, such as borate, could protect it [14,15,16]. It is known that *D*-ribose interacts with many different metal ions [17,18], where some could induce the degradation of ribose and, consequently, ribonucleoside. However, there is no direct evidence for this statement.

Herein, we propose a novel approach for the degradation of biomolecules, such as ribonucleosides, into their respective stable entities (for this case, nucleobases) based on mechanochemical-induced reactions by both metal and inorganic species (nickel and carbonate ions). For this study, the two canonical ribonucleosides guanosine (purine derivative) and uridine (pyrimidine derivative) were used. This strategy can be applied as a model to analyse and interpret astrobiological data from extraterrestrial rocky bodies (meteorites) containing high amounts of carbon (carbonaceous chondrites).

## 2. Results and Discussion

### 2.1. Mechanochemical Degradation Studies of Guanosine and Uridine

**Figure 1:**
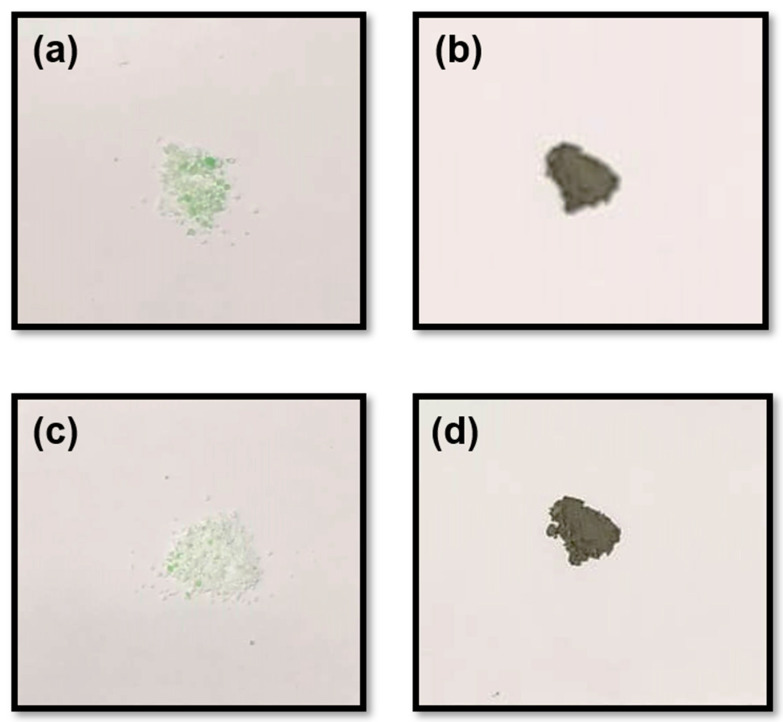
Ribonucleoside-Na_2_CO_3_-NiCl_2_·6H_2_O samples before and after mechanochemical treatment (30 Hz, 6 h): (**a**,**b**) A uridine sample before and after mechanochemical treatment, respectively; (**c**,**d**) A guanosine sample before and after mechanochemical treatment, respectively. Despite the low quality of the pictures, the abrupt change of colours is clear after mechanochemical experiments. These pictures only intend to serve as initial evidence of the reactivity of ribonucleosides.

In this work, purine (guanosine) and pyrimidine (uridine) canonical ribonucleosides were studied. After mechanochemical treatment, the samples containing nickel and carbonate changed from a light green to a very dark brown colour, as shown in Figure 1.

**Figure 2:**
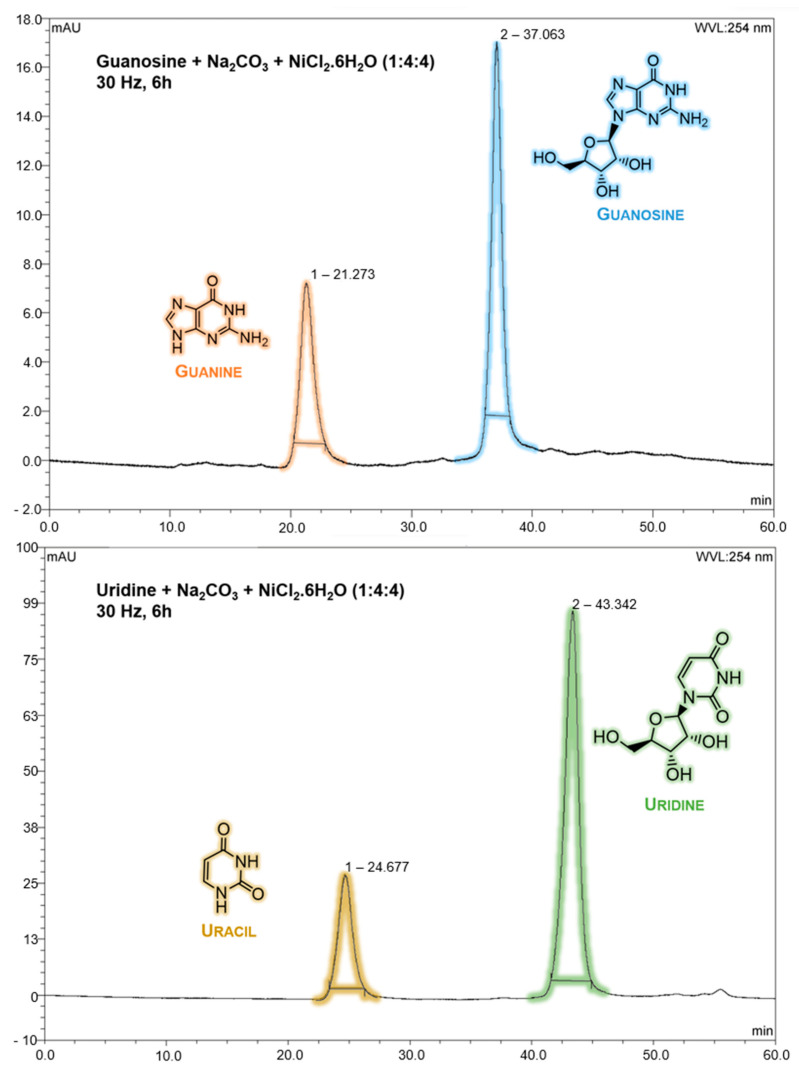
HPLC chromatogram from guanosine and uridine ribonucleosides after mechanochemical treatment (30 Hz, 6 h) with Na_2_CO_3_ and NiCl_2_·6H_2_O on a ribonucleoside-Na_2_CO_3_-NiCl_2_·6H_2_O ratio of 1:4:4. Above: guanine (~21 min, A = 246 and 274 nm) and guanosine (~37 min, A = 253 nm). Below: uracil (~25 min, A = 259 nm) and uridine (~43 min, A = 262 nm). HPLC Run 1 and Run 2 conditions were applied for guanosine- and uridine-treated samples, respectively. Standard nucleobase/nucleoside solutions were previously run to identify their retention times.

After this qualitative result, both samples were analysed by HPLC, and there was direct evidence of the partial ribonucleoside degradation into its respective nucleobase. The retention times for guanine and uracil nucleobases were detected around 21 (A = 246 and 274 nm) and 25 (A = 259 nm) min, respectively, while the retention times for guanosine and uridine ribonucleosides were detected around 37 (A = 253 nm) and 43 (A = 262 nm) min, respectively (Figure 2).

**Figure 3:**
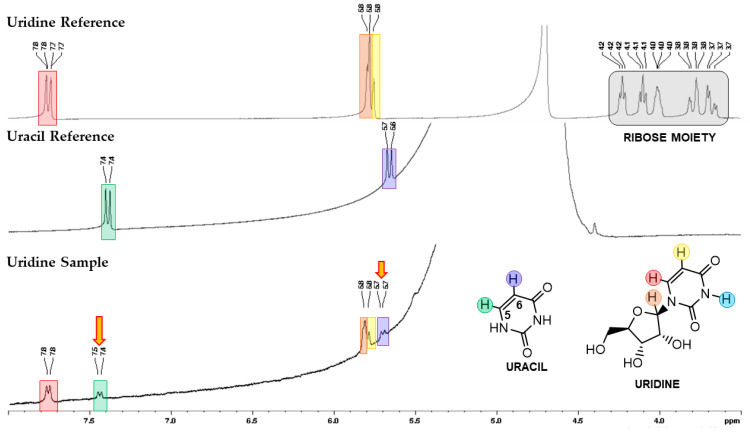
^1^H NMR from a sample containing uridine in H_2_O/D_2_O (9:1) after mechanochemical treatment (30 Hz, 6 h) with Na_2_CO_3_ and NiCl_2_·6H_2_O, in a ratio of 1:4:4 of uridine-Na_2_CO_3_-NiCl_2_·6H_2_O. From the top: a standard of uridine ^1^H NMR spectra in H_2_O/D_2_O (9:1), a standard of uracil ^1^H NMR in H_2_O/D_2_O (9:1), and a treated sample of uridine. Yellow arrows represent the presence of uracil.

Additionally, uracil formation was detected by NMR spectroscopy, confirming the HPLC analysis (Figure 3). From this spectrum, two new signals, C5 (d, ~7.5 ppm, ^1^H) and C6 (d, ~5.7 ppm, ^1^H), corresponding to uracil, were detected. In the case of the guanosine sample, the decreased guanine solubility in D_2_O and DMSO-d^6^ (slightly more soluble than D_2_O) could explain the low ^1^H and ^13^C NMR resolution spectra. These spectra are not shown due to their low quality.

**Figure 4:**
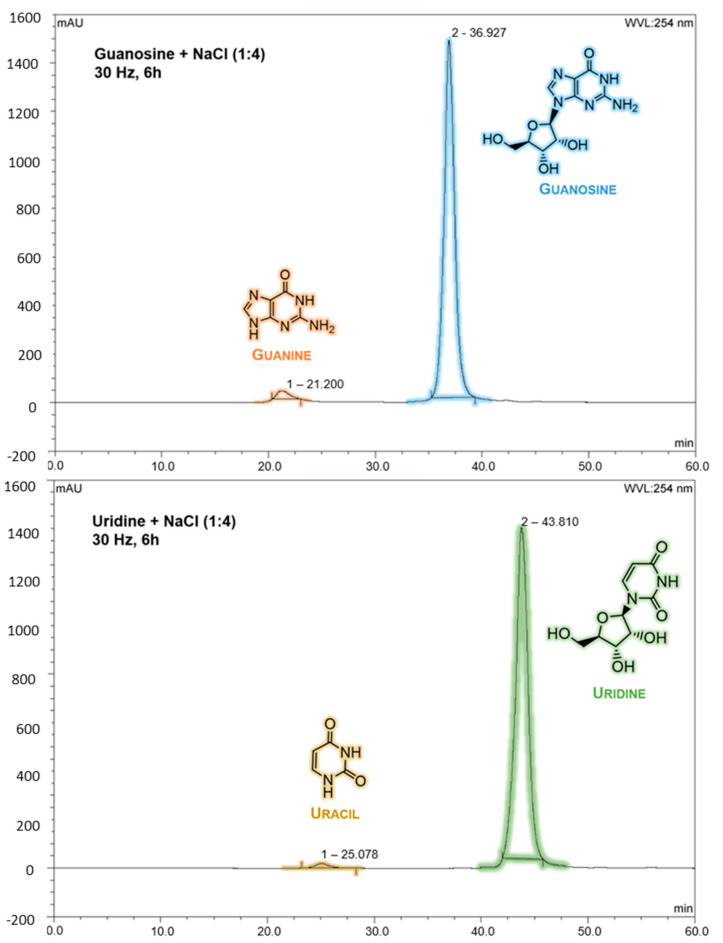
HPLC chromatogram from guanosine and uridine ribonucleosides after mechanochemical treatment (30 Hz, 6 h) with NaCl for a ribonucleoside—NaCl ratio of 1:4. Above: guanine (~21 min, A = 246 and 274 nm) and guanosine (~37 min, A = 253 nm). Below: uracil (~25 min, A = 259 nm) and uridine (~43 min, A = 262 nm). HPLC Run 1 and Run 2 conditions were applied for guanosine- and uridine-treated samples, respectively. Standard nucleobase/nucleoside solutions were previously run to identify their retention times.

Attempts with nickel carbonate (NiCO_3_) were performed, however, without significant results. One important factor that might affect mechanochemical degradation is the availability of both nickel and carbonate ions, which, in the case of NiCO_3_, could not be accomplished in our mechanochemical apparatus (this suggests that higher levels of energy should be required to activate NiCO_3_). Consequently, samples with only “inert ions” (Na^+^ from Na_2_CO_3_ and Cl^−^ from NiCl_2_·6H_2_O) for both ribonucleosides were prepared; however, no substantial degradation was observed in either guanosine or uridine. Figure 4 depicts the HPLC data of guanosine and uridine mechanochemically treated samples with NaCl.

The data from HPLC and NMR spectroscopy clearly demonstrate ribonucleoside degradation, despite the low resolution of the NMR spectra of the guanosine sample. However, the strong change of colour in the original sample of guanosine, together with the HPLC result, supports the degradation of guanosine in guanine. It is worth noting that the reaction conversion is not complete in both samples; however, this can be justified by the large amount of starting ribonucleosides. Quantification was not carried out because the scope of this work was to demonstrate that these types of mechanochemical degradations could have applications for extraterrestrial samples since, during their journey to Earth, they may inevitably face mechanochemical events (such as meteoroid collisions and atmospheric and land impacts).

### 2.2. Ribonucleoside Reactivity and Degradation Mechanism by DFT Calculations

**Figure 5:**
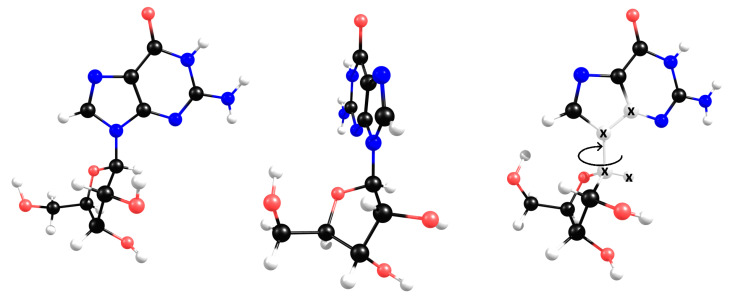
X-ray conformers for guanosine (**left**,**middle**). The definition of the twist angle used for DFT calculations (**right**) based on X-ray experimental twist = 300° (**left**) and twist = 17° (**middle**).

Ribonucleoside structural modelling for DFT calculations requires the probing of the conformational space defined by the ribose moiety, and particularly the glycosyl linkage, to evaluate the thermal availability between the absolute minimum energy conformer and other local minima. In both guanosine and uridine, the *β*-ribose conformation was chosen based on their enantioselectivity occurrence in biomolecules. The glycosyl linkage introduces some conformational complexity due to the possibility of tortional rotation between both ose and nucleobase moieties (Figure 5, right).

**Figure 6:**
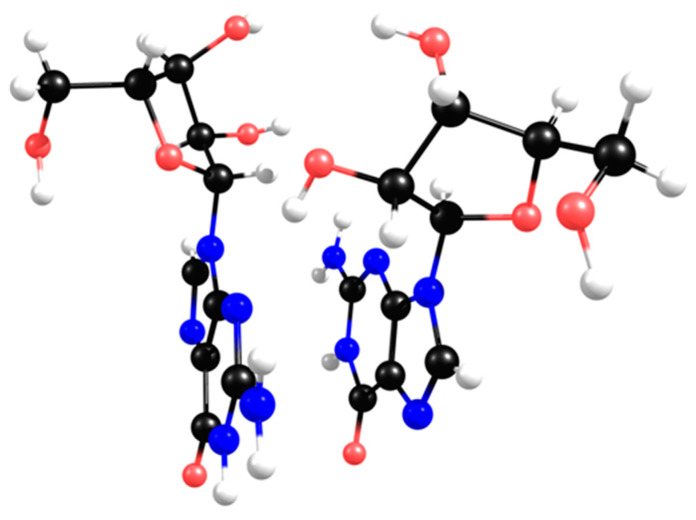
Based on X-ray data, the geometries of the two conformers are π-π-stacked, forming a van der Walls dimer.

The X-ray structure has been determined for guanosine [19] and depicts a π-π-stacked dimer, as represented in Figure 6. Each half of this dimer has a different twist angle around the glycosyl bond, and both were used as starting geometries for DFT calculations (Figure 5, left,middle).

**Figure 7:**
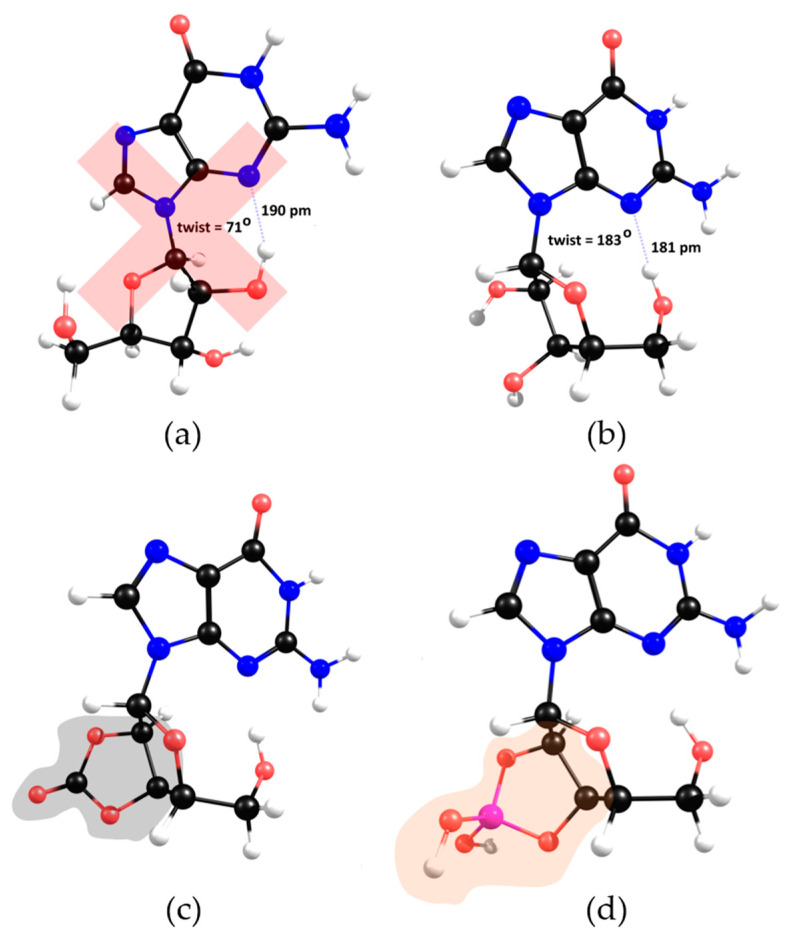
DFT calculations of the most stable conformers of guanosine. A twist angle of 71° enables 2′-hydroxyl and N3 H-bonding (**a**), while a twist angle of 183° enables 5′-hydroxyl and N3 H-bonding (**b**). In the presence of carbonate/borate, the 2′-hydroxyl H-bond is eliminated (**c**,**d**). Grey and orange moieties represent carbonate and borate, respectively.

In the solid state [19], each half of the dimer depicts a different twist angle, and after DFT optimisation, the two stable conformers are within a 10 kJ/mol energy range (Figure 7, upper structures). The most interesting feature of the lower-energy conformers is the existence of a hydrogen bond between a hydroxyl group and the imine nitrogen atom of the nucleobase. However, in the presence of inorganic ions, such as carbonate or borate, the ribose moiety loses its *syn*-hydroxyl groups, breaking the hydrogen bond, which leaves only the methoxy group available for H-binding to the imine nitrogen atom (Figure 7, upper right).

**Figure 8:**
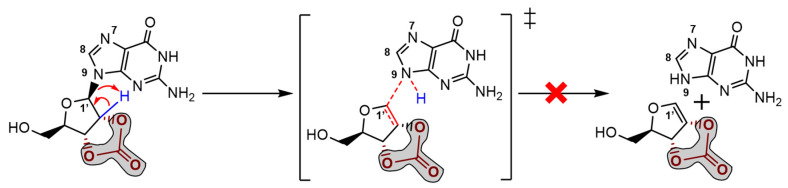
Unsuccessfully proposed guanosine *β* elimination mechanism with nucleobase formation and proton transfer saddle point optimised at HF (Hartree-Fock) level.

The stereochemistry of this specific conformer in borate/carbonate compounds (Figure 7, bottom structures) seems to favour the *β*-elimination of the purine base and the formation of a double bond in the ribose five-membered ring (E_2_ type reaction). The mechanism proceeds through a saddle point in the Potential Energy Surface (PES) resulting from a proton transfer. However, the DFT energetics show that this kind of mechanism is neither kinetically nor thermodynamically viable (Figure 8). Since the nucleobase and the eliminated proton are not in an antiperiplanar position, this might justify the lack of stability of the eliminated product.

**Figure 9:**
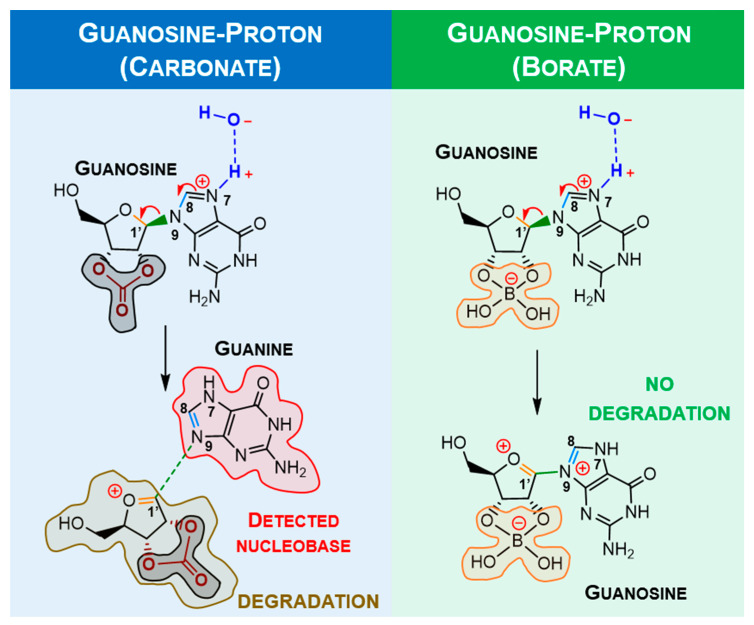
Proposed mechanisms for guanosine degradation and labilisation in the presence of carbonate (blue) or borate ions (green), respectively. In the presence of a proton, the nucleobase moiety is eliminated, forming a double bond in the ribose moiety (highly reactive, which will induce ose degradation). The labilisation of structures promoting nucleobase elimination is lower in ribonucleoside-borate structures. Colours highlight the studied bonds.

Alternatively, it was considered protonation (supplied by water) and a metal ion-assisted mechanism involving positively charged ribonucleosides. Hydrogen could come from water, and nickel was chosen due to its significant levels in delivered extraterrestrial samples. The traditional proton/metal interaction in the furanose oxygen atom followed by a ring opening was found to be highly unfavourable when compared with the similar binding of guanosine (*N7* position). Some selected optimised bond lengths are shown in Table 1 to provide evidence of labilisation (promoted by the stretching) of the glycosylic linkage in carbonate as opposed to borate. The observed bond shortening/lengthening is compatible with the strengthening evolution depicted in Figure 9 and summarised in Table 1.

**Figure 10:**
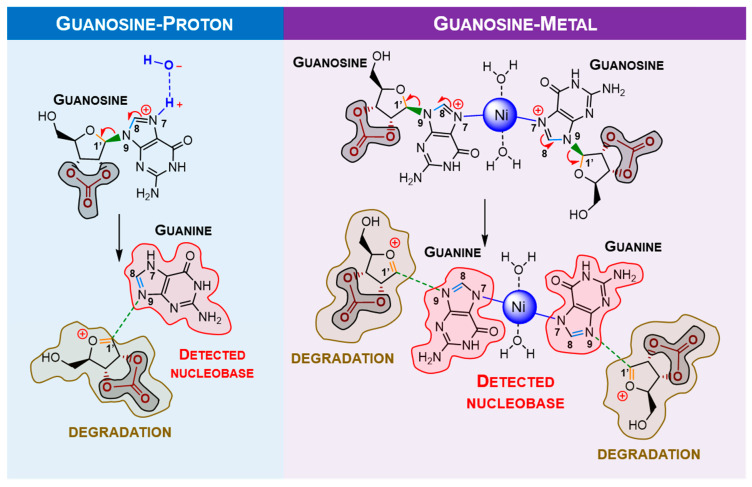
Proposed mechanism for mechanochemical degradation of guanosine, promoted by nickel (II). In the presence of a proton/metal, the nucleobase moiety is eliminated, forming a double bond in the ribose moiety (highly reactive, which will induce ose degradation). However, this mechanism suggests that the metal ion could catalyse two ribonucleosides simultaneously, being more effective than the proton. Carbonate acts as the “activator” for ribonucleoside degradation.

From Table 1, it is noticeable that the stretching of N9-C1′ and the shortening of C1′-O are more significant with carbonate than with borate, which implies that carbonate is more suitable as an “activator” compared with borate. Since the degradation reaction containing borate bound to the sugar moiety should not happen, metal complexation with guanosine was only calculated for the carbonate moiety (Figure 10) and summarised in Table 2.

From Table 2, in the presence of nickel, the formation of double bonds within the guanine moiety C8-N9 and ribose derivative structure C1′-O and stretching of N9-C1′ is notable, which corresponds to the proposed degradation model. By comparing it with proton catalysis, it seems that labilisation of the aforementioned selected bonds is less intense. However, as proposed in the degradation mechanism, the nickel ion might interact with two ribonucleosides, making it a more efficient mediator of the reaction.

**Figure 11:**
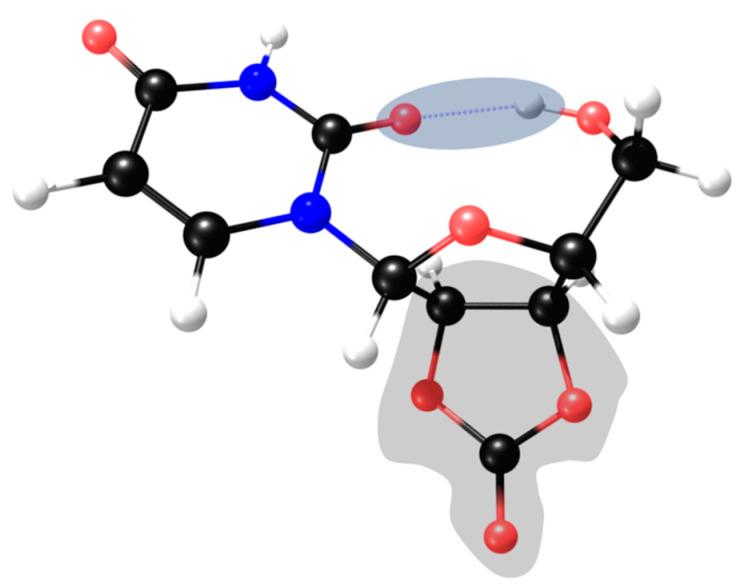
DFT calculated neutrally optimised conformers of uridine. Similar to guanosine, 5′-hydroxyl and O2 H-bonding are the predominant H-bond stabilisations (blue). The carbonate moiety is highlighted in grey.

**Figure 12:**
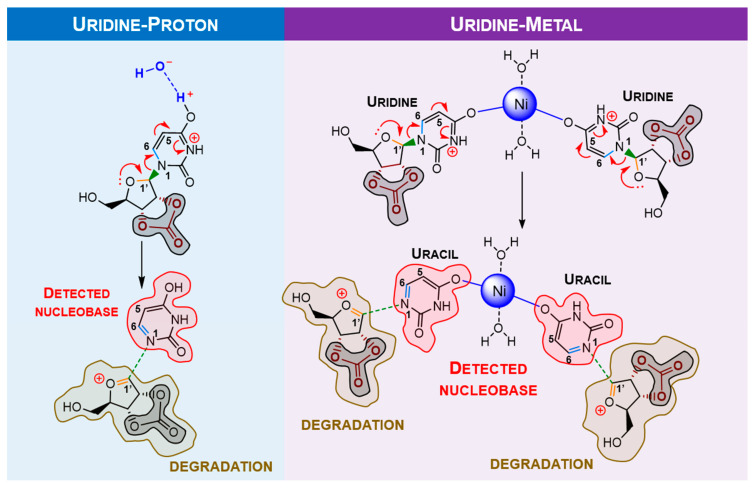
Proposed mechanism for mechanochemical degradation of uridine, promoted by nickel (II). In the presence of a proton/metal, the nucleobase moiety is eliminated, forming a double bond in the ribose moiety (highly reactive, which will induce ose degradation). Similar to guanosine degradation, the metal ion contribution is more effective than proton catalysis. Carbonate acts as the “activator” for ribonucleoside degradation.

To study the observed behaviour with pyrimidine analogues, calculations for uridine were carried out. Assuming the “protective” behaviour of borate, uridine calculations were developed considering the carbonate moiety. In this case, the most stable conformer is also defined by a H-bond to the hydroxyl of the ose moiety (Figure 11). Proton and metal effect represented in Figure 12.

From Table 3, identically to guanosine, there is evidence of nucleobase elimination with double bond formation on the ribose moiety. Similar to the abovementioned results, bond labilisation is slightly increased by protonation rather than metalation; however, like guanosine, the nickel ion can bond to two uridines, making nickel more effective for nucleobase elimination. Comparing guanosine with uridine ribonucleoside, it is clear that labilisation of uridine bonds is much more accentuated. Either way, as mentioned in Section 1, uracil is thermally more stable than guanosine and, hence, less reactive. The change in reactivity might be related to the presence of carbonate (an activator), which increases ribonucleoside reactivity. Moreover, the applied mechanochemical energy might also be related to this outcome, which can easily surpass the activation barrier for uridine degradation. In addition to that, the C1′-O double bond formation in both ribonucleosides implies that, upon nucleobase separation, the ribose moiety reactivity increases towards its decomposition, which could justify the absence of free-ribose in both NMR spectroscopic and HPLC data. As a sum, the DFT calculations corroborate mechanochemical experiments.

## 3. Materials and Methods

In this experimental work, all the reagents were handled without further purification, including guanosine (Sigma-Aldrich, St. Louis (Missouri), EUA, 98%), uridine (Alfa Aesar, Lancashire, UK, 99%), guanine (Sigma-Aldrich, 98%), uracil (Alfa Aesar, 99%), sodium carbonate (J. T. Baker, Phillipsburg, EUA, 99.7%), nickel chloride hexahydrate (Sigma-Aldrich, 98%), and sodium chloride (NaCl, Panreac, Barcelona, Spain, 99.5%).

### 3.1. Milling Process

To mimic shock/impact reactions, a mixer mill was used. Reactants were pre-ground using mortar and pestle, dried, and then ground in a Retsch MM400 ball mill (operated at 30 Hz) for a total time of 6 h (four cycles of 1.5 h). Two 7 mm balls were added to each 10 mL stainless steel reactor. The dry neat-grinding studies were carried out with (1) a mixture of ribonucleoside (guanosine or uridine) nickel chloride, NiCl_2_·6H_2_O, and sodium carbonate, Na_2_CO_3_, in a 1:4:4 ratio (for a total weight of 180 mg), respectively (samples were dried for 5 min at 90 °C prior to the mechanochemical treatment); or (2) a mixture of ribonucleoside (guanosine or uridine) and sodium chloride, NaCl, in a 1:4 ratio to verify the contribution of this salt for the degradation of ribonucleosides (for a total weight of 180 mg). Previous calculations were performed for each case to achieve the described ratio. For all reactions, at least 100 mg were used for milling processes.

### 3.2. HPLC Analysis

The samples were also analysed on an UltiMate™ 3000 Standard (SD) HPLC, composed of a quaternary pump LPG-3400SD, an autosampler WPS-300SL, and a column oven TCC-3000SD coupled in-line to a diode-array detector, DAD-3000. Aliquots were injected into the column via a Rheodyne injector with a 100 μL loop in the in-line split-loop mode. Separations were conducted with a Luna C18 100 Ǻ (250 × 4.6 mm, 5.0 μm) at 40 °C using a flow rate of 0.3 mL·min^−1^, a mobile phase of formic acid 0.1% at pH 4.00 (eluent A), and acetonitrile (eluent B), using two different conditions: Run 1—99% A and 1% B (0–25 min), 95% A and 5% B (25–55 min), and 99% A and 1% B (55–60 min); Run 2—100% A (0–10 min), 99% A and 1% B (10–20 min), 98% A and 2% B (20–30 min), 95% A and 5% B (30–50 min), and 100% A (50–60 min). The previous conditions are optimised for all canonical nucleobase and nucleoside separations. Blank tests with purchased canonical nucleobases and ribonucleosides were carried out to identify the corresponding retention time after mechanochemical experiments.

### 3.3. NMR Spectroscopy Analysis

^1^H nuclear magnetic resonance spectroscopy spectra were acquired in a 400 MHz Avance III Bruker Ultra Shield spectrometer equipped with a 5 mm BBO probe at room temperature by using a mixture of water and deuterium oxide (D_2_O, Eurisotop, 99.9%, δ = 4.79) or dimethyl sulfoxide-d^6^ (DMSO-d^6^, Eurisotop, 99.8%, δ = 2.5) as solvents. The spectra were analysed on TopSpin 3.6.4 software (academic license). In all samples, sonochemistry with Transsonic T460 Elma ultrasound equipment was used due to solubilisation constraints.

### 3.4. Theoretical Calculations

All theoretical calculations were of the density functional theory (DFT) type and carried out using the General Atomic and Molecular Electronic Structure System (GAMESS-US) version R3. B3LYP [20] functional was used in ground state calculations, while the range-separated Coulomb Attenuated Method-B3LYP (CAM-B3LYP) was used in the excited state time-dependent density functional theory (TDDFT) calculations. A 6-31G** basis set was used in either DFT or TDDFT calculations. The saddle points for transition states were optimised using Hessians, calculated by the Couple Perturbed Hartree Fock Method, and the negative eigenvalue following the algorithm. The optimised geometries were checked for the appropriate number of zero/negative eigenvalues. Mechanistic studies were based on a full vibrational analysis of the optimised geometries for products and reactants. The vibrational modes were visually analysed, and the one that resembles the mechanistic Internal Reaction Coordinate (IRC) was chosen and perturbed in the direction of the saddle point to obtain a single negative eigenvalue in the Hessian. Subsequently, this was followed and optimised to the nearest saddle point. The single imaginary frequency and eigenvector were computed at the saddle point to confirm they parallel the proposed IRC.

## 4. Conclusions

In this study, ribonucleoside degradation with nucleobase preservation was observed with low shock/collision energy in the presence of nickel(II) and carbonate ions. From NMR and HPLC data, it is possible to confirm the presence of starting ribonucleoside, which indicates that some starting material did not react. The presence of free-ribose characteristic signals was not detected in both NMR and HPLC data, which goes along with the mechanism predictions obtained by DFT calculations. Nevertheless, under these experimental conditions, ribose degradation is still plausible. The scope of this work was to demonstrate the viability of these reactions. DFT calculations demonstrate that reactivity towards uridine is slightly higher than with guanosine, which contradicts the thermal stability of the corresponding ribonucleosides. However, several other factors, such as the contribution of carbonate and mechanical energy, might justify this outcome.

These results imply that environments with high mechanochemical activity might contribute to the degradation of organic content, where metals and/or inorganic ions could also have an active role. Mechanochemical degradation of polymers and pollutants is already studied [21]; however, further applications in the areas of prebiotic chemistry and geochemistry might be worth exploring. Mechanochemistry was already used to mimic the meteorite-catalysed synthesis of biomolecules [22,23]; however, it was never applied to the degradation of extraterrestrial organic molecules. The formation of asteroids, meteoroids, and meteorites is one good example of extreme mechanochemical activity [24,25,26], which, allied with the high chemical complexity of samples, could promote the degradation of several organic molecules. Minerals containing both nickel and carbonate have been detected among meteorites; however, it has not yet been confirmed whether their origin is from sample weathering [27,28]. Nevertheless, minerals containing cationic nickel have also been detected [29,30]. We identify nickel as a mediator of degradation reactions by mechanochemistry, and this capacity is related to nickel being a good Lewis acid. The other contributor is carbonate, a derivative of carbon dioxide (a stable compound that could have originated from the degradation of organic matter), which acts as an activator for ribonucleoside degradation. However, the degradation of biomolecules is directly related to the complexity of the extraterrestrial sample; therefore, many chemical entities may contribute to its degradation. On the other hand, from DFT predictions, the proton was slightly more reactive than nickel; however, the concentration of the first in extraterrestrial samples, compared with the latter, is far less.

Another source of mechanochemical activity that was never explored is the effect of planetary atmospheres on the organic matter of extraterrestrial samples. Usually, only the inorganic content of meteoroids and meteorites (which result in physical and chemical transformations) is analysed, often neglecting the organic content. For instance, meteoroids, when coming across the Earth’s atmosphere, often suffer fragmentation, which can be the source of major mechanochemical transformations. Understanding the relationship between atmospheric composition and shock wave energy could give us new insights into the exogenous delivery of organic content.

This unexplored approach, based on degradation reactions, could give us an additional comprehension of the astrobiological results of meteorites and returned samples collected in extraterrestrial environments, as well as the prebiological evolution of life. Nevertheless, the applied mechanochemical energy in these experiments is nowhere near real shock/collision scenarios; therefore, it is necessary to correlate the levels of energy given to the mechanochemical apparatus with the real collision/impact events. Moreover, it would be worth exploring other types of mechanochemical systems to be able to come closer to the real conditions. It is important to note that there are other types of degradation pathways in extraterrestrial environments, which the proposed model does not exclude. For instance, thermal degradation (originated from friction) and radiative degradation (a consequence of the Sun’s radiative pressure) are two major pathways for the degradation/modification of extraterrestrial organic molecules. Despite that, mechanochemistry has the advantage of being able to propagate “homogeneously” throughout the entire sample, while thermal and radiative pathways are mainly superficial [25]. Either way, the several energy sources should be explored individually but also as a whole in order to survey possible degradation patterns.

In other words, with this work, we propose that some organic molecules found in meteorite samples might not be directly synthesised in an extraterrestrial environment but rather be the degradation product of a more complex molecule. As an example, nucleobases present in meteorite samples could be the biosignature of extraterrestrial ribonucleosides. RNA nucleobases and ose moieties were already detected in carbonaceous chondrite meteorites [31,32,33]; however, direct condensation of ribose and nucleobases has not yet been obtained from a prebiotic standpoint [34,35,36]. Therefore, this might suggest that nucleobases can be the degradation product instead of the starting material for ribonucleoside prebiological synthesis. In the future, it is worth exploring other nucleosides to assess the reproducibility of the degradation model. In addition, it is also important to study if there is any type of interconversion between nucleosides and nucleobases, which would help explain the distribution of nucleobases in meteorites and asteroids. Since extraterrestrial samples are known for their complexity, in the future, it would be worth exploring the proposed mechanochemical reactions with different metals that are also abundant in meteorites, focusing on those that exhibit strong Lewis acid activity.

As referred to in Section 1, biological covalent-bond-type mechanophores were not yet found within biological systems. Since guanosine and uridine are very important building blocks in our modern biological systems, we could consider the *N*-glycosidic bond as a hypothetical “covalent-bond type mechanophore”. By studying its stability towards mechanical energy, we could understand how molecules containing “weak N-C bonds” could (or not) survive high mechanochemical activity environments. The presented model is not restricted to ribonucleosides; hence, a variety of soluble and insoluble extraterrestrial molecules should be explored, such as amino acids, sugars, and polyaromatic hydrocarbons, respectively.

## Figures and Tables

**Table 1:** Selected bond lengths (pm) around the glycosylic bond in guanosine and bond reorganisation upon protonation at *N*7 for carbonate and borate moieties. Colours highlight the studied bonds.

Bond	Carbonate (CO_3_^2−^)		Borate (BO_3_^3−^)	
	Neutral	Protonate	Neutral	Protonate
C8-N9	138.1	135.0	138.1	134.9
N9-C1′	147.2	148.4	147.2	146.5
C1′-O	141.5	139.0	141.5	139.7

**Table 2:** Selected bond lengths (pm) around the glycosylic bond in guanosine and bond reorganisation upon metalation at N7 and comparison with proton catalysis. Colours highlight the studied bonds.

Bond	Neutral	Proton (H^+^)	Metal (Ni^2+^)
C8-N9	138.1	135.0	136.1
N9-C1′	147.2	148.4	148.1
C1′-O	141.5	139.0	139.2

**Table 3:** Selected bond lengths (pm) around the glycosylic bond in uridine and bond reorganisation upon protonation and metalation at *N*3. Colours highlight the studied bonds.

Bond	Neutral	Proton (H^+^)	Metal (Ni^2+^)
C6-N1	138.6	135.1	135.3
N1-C1′	146.4	149.9	149.8
C1′-O	140.6	138.5	138.5

## Data Availability

The data produced in this work is present within this paper.
